# Case Report: mRNA vaccination-mediated STAT3 overactivation with agranulocytosis and clonal T-LGL expansion

**DOI:** 10.3389/fimmu.2023.1087502

**Published:** 2023-02-02

**Authors:** Julia R. Hirsiger, Alexandar Tzankov, Ilaria Alborelli, Mike Recher, Thomas Daikeler, Stefani Parmentier, Christoph T. Berger

**Affiliations:** ^1^ Translational Immunology, Department of Biomedicine, University of Basel, Basel, Switzerland; ^2^ Institute for Pathology, University Hospital Basel, Basel, Switzerland; ^3^ University of Basel and ETH Zurich, Botnar Research Centre for Child Health, Basel, Switzerland; ^4^ Pathology, Institute of Medical Genetics and Pathology, University Hospital, Basel, Switzerland; ^5^ Primary Immunodeficiency, Department of Biomedicine, University of Basel, Basel, Switzerland; ^6^ University Center for Immunology, University Hospital Basel, Basel, Switzerland; ^7^ Rheumatology Clinic, University Hospital Basel, Basel, Switzerland; ^8^ Department of Hematology, Claraspital Basel, Basel, Switzerland

**Keywords:** T-LGL, STAT3, IL-6, neutropenia, COVID-19, mRNA vaccine, TLR (Toll like receptors), TLR3

## Abstract

Vaccines against SARS-CoV-2 are the most effective measure against the COVID-19 pandemic. The safety profile of mRNA vaccines in patients with rare diseases has not been assessed systematically in the clinical trials, as these patients were typically excluded. This report describes the occurrence of agranulocytosis within days following the first dose of an mRNA-1273 vaccination against COVID-19 in a previously healthy older adult. The patient was diagnosed with a suspected STAT3 wild-type T-cell large granular lymphocytic leukaemia (T-LGL). Neutropenia was successfully treated with IVIG, glucocorticoids, and G-CSF. In vitro experiments aimed at elucidating the pathways potentially causing the mRNA vaccine-associated neutropenia indicated that the mRNA, but not the adenoviral Ad26.COV2.S vector vaccine, triggered strong IL-6/STAT3 activation in vitro, resulting in excessive T-cell activation and neutrophil degranulation in the patient but not in controls. mRNA-1273 activated TLR-3 suggesting TLR mediated IL-6/STAT3 pathway activation. To complete the primary series of COVID-19 immunization, we used a single dose of Ad26.COV2.S vector vaccine without reoccurrence of neutropenia. The T-LGL clone remained stable during the follow-up of more than 12 months without ongoing therapy. Our data suggest that switching the immunization platform may be a reasonable approach in subjects with rare associated hematologic side effects due to excess STAT3-mediated stimulation following mRNA vaccination. Using in vitro testing before re-administration of a (COVID) vaccine also has relevance for other rare immune events after (mRNA) vaccination.

## Introduction

Large granular lymphocyte (LGL) leukaemia is a rare lymphoproliferative disease of the mature T cell or natural killer lineages (1). Patients can be asymptomatic, present with non-specific symptoms, or with neutropenia or neutropenic fever (2). Autoimmune diseases, most prominently rheumatoid arthritis (RA), have been associated with expansions of T-LGL clones or T-LGL leukaemia. In T-LGL cohorts, up to 26% of patients also have RA (2, 3), and in RA cohorts, T-LGL clones can be found in about 3.6% of the patients (4). T-LGL pathogenesis involves resistance to activation-induced cell death due to constitutive activation of the signal transducers and activators of transcription 3 (STAT3), providing survival signals to the T-cell clones (5). In many cases, STAT3 gain-of-function (GoF) mutations are found (6). In CD8 T-LGL, about 40% carry a STAT3 GoF mutation (7), but frequencies vary from 11-75% amongst cohorts (6). In contrast, CD4+ T-LGL is rarely associated with a STAT3 GoF mutation, but 15-55% of CD4+ T-LGL patients carry STAT5b mutations (6). Interleukin-6 (IL-6) signals *via* STAT3 and inhibits apoptosis in T-LGL (8). T-LGL populations may expand during infections (9–12) or after transplantation (13).

During the COVID-19 pandemic, novel mRNA vaccines were approved. The mRNA can cause activation of innate immune sensors, including toll-like receptor-3 (TLR-3) (14–16). TLR-3 activation induces IL-6/STAT3-activation. Subjects with immune diseases were excluded from the clinical mRNA vaccine trials. Hence, safety assessment in such patients depends on post-marketing vaccine safety surveillance and case reporting.

## Methods

The local ethic commission approved the study (Ethikkommission Nordwest- und Zentralschweiz (EKNZ 2015-187)), and the patient provided written informed consent. Immunophenotyping was performed on peripheral blood mononuclear cells (PBMC) using: CD3 (AF^®^700, OKT3), CD4 (BV510™, SK3), CD8 (Pe-Cy7, SK1), TCRab (AF^®^488, IP26), CD57 (APC, HNK-1), CD95 (PE, DX2), and CD69 (BV785™, FN50). All samples were acquired on a BD LSRFortessa and analyzed using FlowJo v10.8.1.

For *in vitro* stimulations, we used mRNA-1273 [1:200] (Moderna^®^) or Ad26.COV2.S [1:200] (Janssen) on cells with or without 30 minutes pre-incubation with siltuximab [0.01-1.0 μg/ml] or tocilizumab [30μg/ml] for IL-6 blocking experiments, or IVIG [30μg/ml] as a control. For immuno-stainings assessing phosphorylation of STAT3 and TLR3, permeabilization was performed with Perm III buffer (BD Biosciences) and the following antibodies were used: anti-human pSTAT3 (Cell Signaling) or anti-human pTLR3 (Tyr759) (Invitrogen) (Cell Signaling) followed by anti-rabbit IgG detection antibody (AF^®^647 conjugate, Invitrogen). For intracellular cytokine stainings we used antibodies against IFNγ (APC, 4S.B3). The interferon-stimulated gene MX1 and IL-6 were quantified by qPCR. Reverse-transcription PCR was performed with GoTaq qPCR Master Mix in triplicates in a 7500 Fast Real-Time PCR System (Applied Biosystems). Genes were quantified by qPCR using the following primers: MX1 fwd: 5’-TTAACCTCCACAGAACCGCC-3’, rev: 5’-GCGGAT CAGCTTCTCACCTT-3’, IL-6 fwd: 5’- ACTCACCTCTTCAGAACGAATTG-3’, rev: 5’-AGCCATCTTTGGAAGGTTCAG-3’. qPCR data were normalized to two reference genes (GAPDH and PGK1) using previously described methods (17). Gene expression was normalized to the mean of n=5 healthy controls, and the fold change to the controls determined using the 2^−ΔΔCt^ method.

Gene sequencing for pathogenic STAT3 or STAT5b mutations was done on ​​the Ion Torrent S5 platform using a validated customized Oncomine Lymphoma Panel (for details, see supplementary methods) (18).

## Results

### Clinical presentation of agranulocytosis following mRNA-1273 vaccination

**Figure 1:**
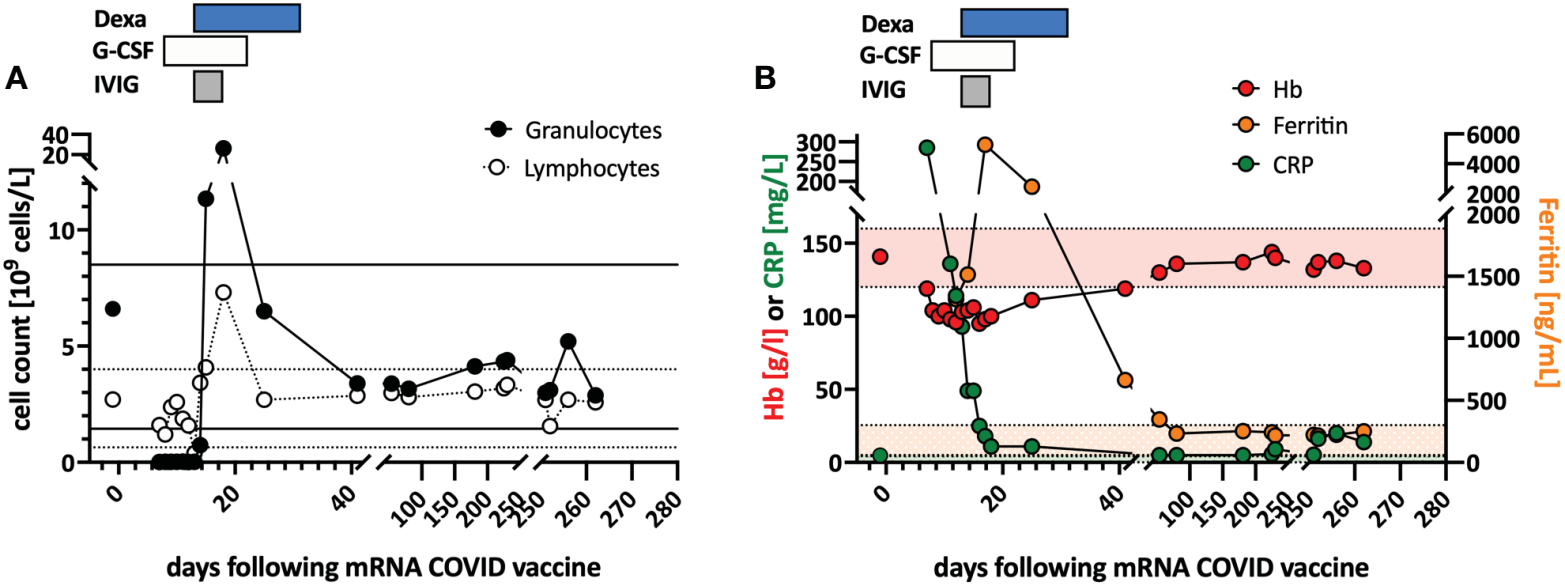
Clinical course and *in vitro* stimulation with mRNA-1273. **(A)** Longitudinal peripheral leucocyte and lymphocyte counts are shown. The black lines indicate the normal reference range of the granulocyte count, and the dotted lines the reference range for the lymphocyte count. **(B)** Longitudinal measurements of hemoglobin (red), c-reactive protein (CRP; green), and ferritin (orange) serum levels as markers of systemic inflammation are indicated. Shaded areas indicate the reference range for the markers (color coded). Treatments are indicated at the top of the graphs: DEXA, dexamethasone; G-CSF, granulocyte stimulating factor; IVIG, intravenous immunoglobulin.

**Figure 2:**
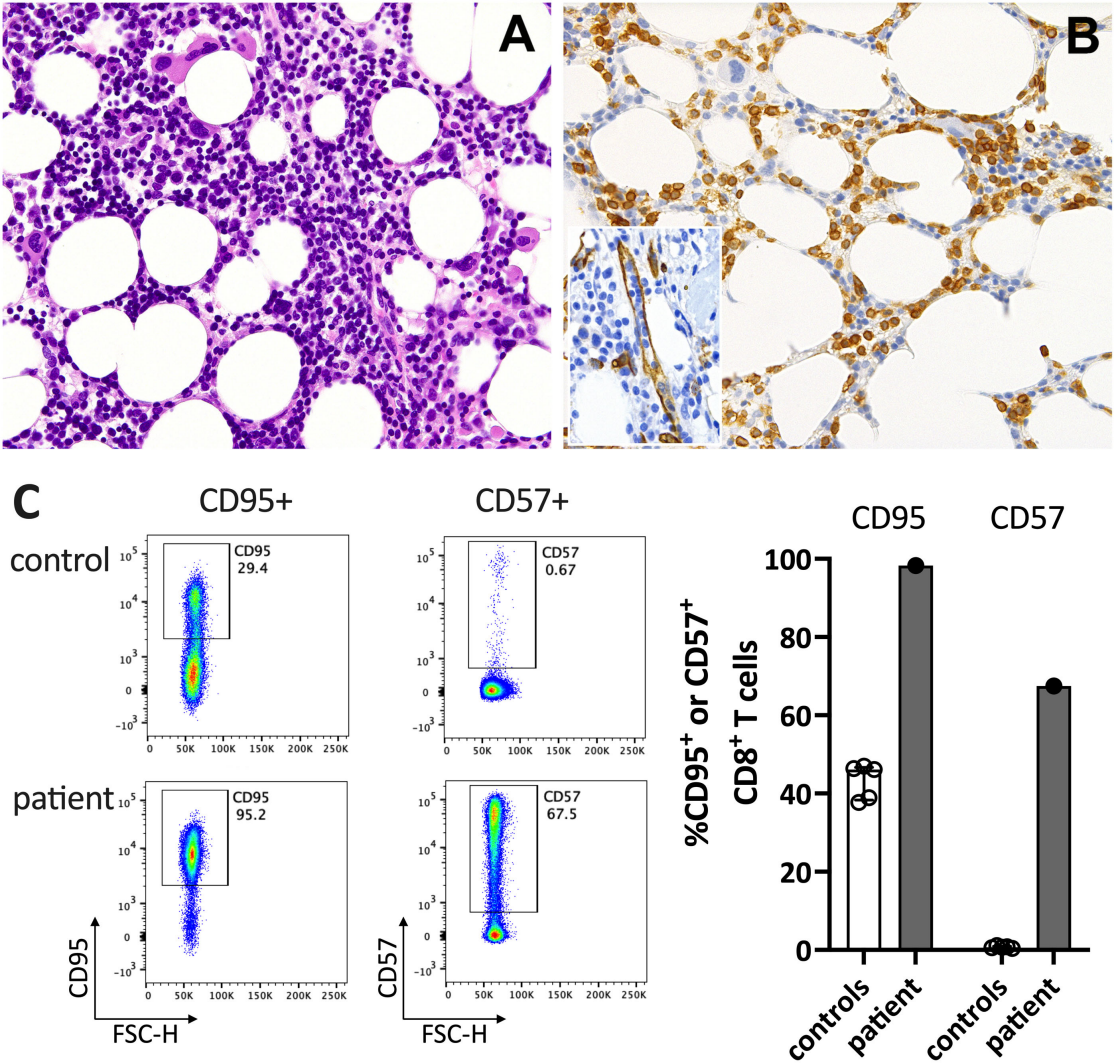
Immunohistochemistry of the bone marrow compatible with T-LGL. **(A)** H&E staining shows a hypercellular bone marrow with slightly increased and left-shifted megakaryopoiesis, substantially reduced and left-shifted myelopoiesis, and interstitial lymphocytosis. **(B)** Immunohistochemistry for anti-CD3 (T cells) confirmed significant interstitial T-cell lymphocytosis with some linearity and intravascular spread (shown as an insert in the figure; anti-CD34 was used to mark the vessels). Magnification 400x (CD3) and 630X (insert; CD34). **(C)** Flow cytometry plots (left) and data summary graph (right) show high CD95 and CD57 expression on T cells of the patient compared to healthy controls (ctrl; n=5). FSC-H= forward scatter height.

In June 2021, a previously healthy woman in her 60s was referred with neutropenic fever. She had fatigue and malaise, starting 24 hours following the first dose of the COVID-19 vaccine mRNA-1273. On the sixth day post-vaccination, she developed fever (39°C), sore throat, coughing and vomiting. She denied taking non-steroidal anti-inflammatory drugs that could cause neutropenia (i.e. NSAIDs or metamizole). She is a non-obese, non-smoker and has no diabetes or hypertension. The only available blood count from four years earlier was normal. The physical examination was unremarkable. Specifically, it revealed no palpable lymph nodes or splenomegaly and no petechia, rash, or pharyngitis. The CT scans showed several marginally increased cervical lymph nodes without organomegaly, especially without splenomegaly. Systemic inflammation markers were very high with a C-reactive protein (CRP) of 285 mg/l (reference <5mg/l) and a ferritin peak level of 5276 ng/ml (reference <300ng/mL), compatible with macrophage activation (**Figure 1**). Tests for SARS-CoV2 and other infections (blood cultures, multiplexed PCR panels from stool and nasal swabs, serology for hepatitis viruses, CMV, and Parvovirus B19) were negative. PET-CT revealed pronounced diffuse hypermetabolism of the hematopoietic bone marrow and spleen. Bone marrow biopsy showed infiltration of CD8^+^ T cells and hypercellularity with severely reduced granulopoiesis, consistent with pure white cell aplasia (**Figure 2A**). Interstitial T-cell lymphocytosis with some linearity and intravascular spread of the T cells was suggestive of T-LGL (19) (**Figure 2B**). Immunophenotyping of the lymphocytes in the peripheral blood and bone marrow identified a large CD8+ T cell population (~45% of all T cells) expressing CD5^dim^, CD7, CD16^partial^, TCRαβ, HLA-DR and that was negative for CD56, TCRγδ, CD30, CD10, CD25, and PD1 in the clinical routine lab, compatible with T-LGL. The T- and B-cell clonality and translocation analysis (20) showed a dominant TCRγ-rearrangement with a base pair length of 200 bp (type V-I) on a polyclonal background. There was no evidence of B-cell clonality or translocations (t(11;14), t(14;18)). Next-generation sequencing applying a custom lymphoma panel covering 68 genes commonly mutated in lymphomas (21, 22) identified no mutation, particularly not in *STAT3* or *STAT5b*. A diagnosis of possible T-LGL leukemia with pure white cell aplasia was made.

Treatment with a broad-spectrum antibiotic and recombinant human G-CSF was initiated, but peripheral agranulocytosis persisted. Only after treatment with intravenous immunoglobulins (IVIG 30g; given in four doses) and dexamethasone (16mg/day) was started agranulocytosis and systemic inflammation instantly resolved (**Figure 1**), and after two weeks, she recovered completely. During follow-up, the T-LGL population declined to about 34% of all T cells. No further treatment was initiated as she remained asymptomatic.

In January 2022, she was referred to our immunology clinic for counseling regarding completing the COVID-19 immunization series. She had a tonsillectomy for recurrent tonsillitis as a young adult. She never experienced an episode of cytopenia or lymphocytosis prior to the here described event, and had no history of autoimmunity or opportunistic infections. Detailed patient history revealed mild oral sicca and recurrent arthralgias but no arthritis. Anti-nuclear antibodies (ANA), anti-neutrophilic cytoplasmatic antibodies (ANCA), rheumatoid factor and anti-citrullinated protein antibodies (ACPA) were negative. She had been vaccinated according to the Scottish immunization plan and never experienced relevant side-effects from a vaccine. Her sister has rheumatoid arthritis and her brother retroperitoneal fibrosis.

At his time, the clinical exam was unremarkable. She had no lymphadenopathy or splenomegaly. The white blood count (WBC) and CRP were normal. Anti-SARS-CoV-2-N(ucleoprotein)-IgG/IgM were negative and anti-S(pike)-IgG/IgM at low levels (61.8 U/mL (<0.7); Roche Elecsys^®^ assay). The T-LGL clone was stable at about 800/ul. The CD8^+^ T cells expressed high levels of CD95 and CD57, typical for T-LGL (**Figure 2C**).

### mRNA-1273 mediated IL-6/STAT3 pathway activation *via* TLR stimulation

**Figure 3:**
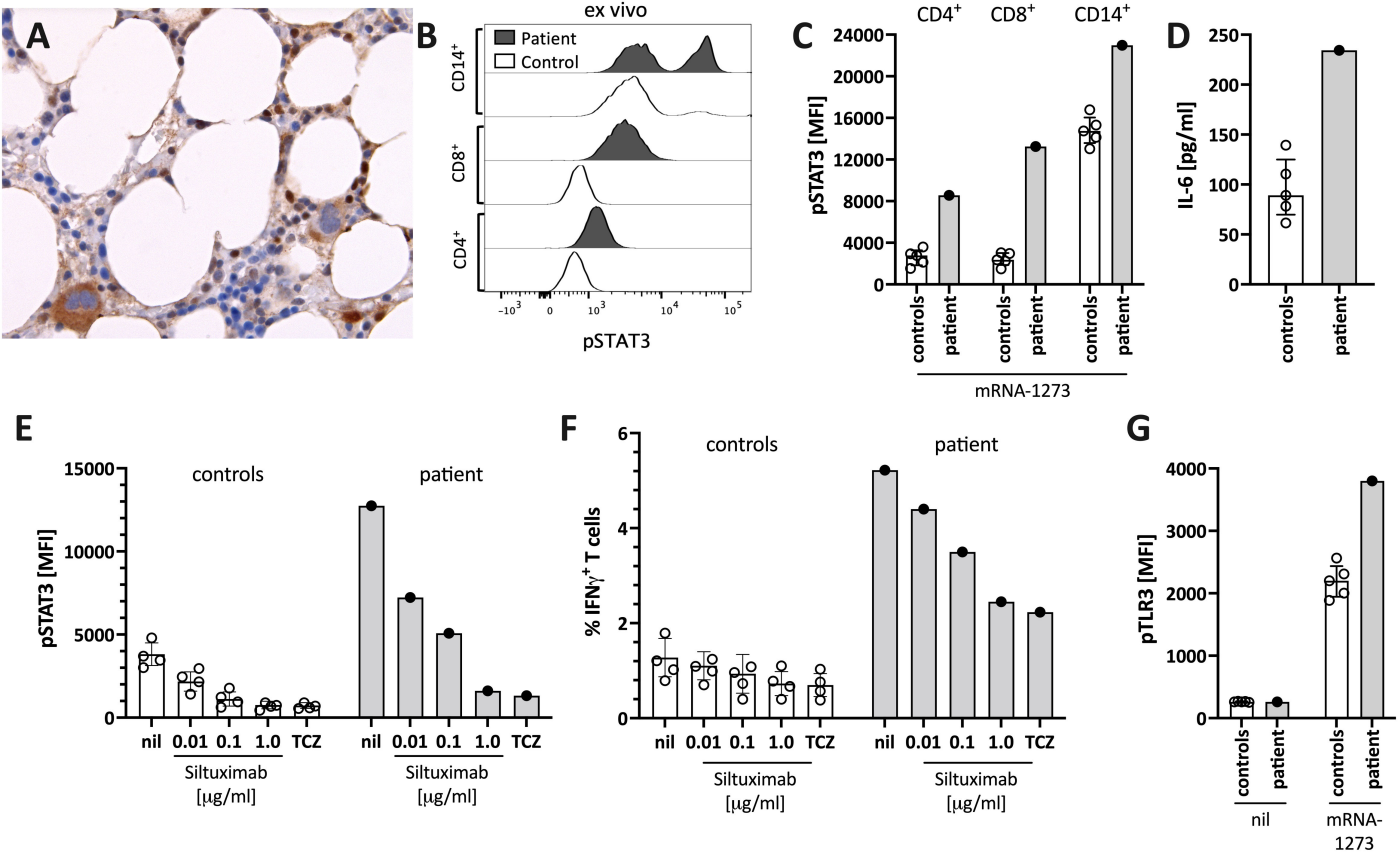
Enhanced STAT3/IL-6 pathway activation in the bone marrow and *in vitro* by mRNA-1273 stimulation. **(A)** Immunohistochemical staining for phosphorylated-STAT3 (pSTAT3) showing scattered positive interstitial cells, morphologically consistent with lymphocytes, suggestive of STAT3 activation at diagnosis; note the negatively staining erythropoiesis and the nuclear negativity of the megakaryocytes. Immunoperoxidase staining; original magnification 630x. **(B)** Histogram showing pSTAT3 in T cells (CD4^+^ and CD8^+^) and monocytes (CD14^+^) directly *ex vivo*. **(C)** STAT3 phosphorylation (pSTAT3) following *in vitro* stimulation of peripheral blood immune cells with the mRNA-1273 vaccine. **(D)** Supernatants of PBMC stimulated with mRNA-1273 show higher IL-6 production (ELISA). **(E)** mRNA vaccine induced *in vitro* STAT3 phosphorylation was dose-dependently blocked by the anti-IL-6 antibody siltuximab and the IL-6 receptor blocker tocilizumab (TCZ; used at 30 μg/ml, which corresponds to the serum concentration of treated subjects (see also **Supplementary Figure S2**). **(F)** mRNA stimulation induced T cell activation, as indicated by IFNγ positive T cells, was inhibited by IL-6 blockade (siltuximab or TCZ). **(G)** mRNA-1273 stimulation resulted in TLR3 phosphorylation in monocytes of healthy controls, and even more pronounced of the patient. MFI= median fluorescence intensity in flow cytometry. nil= only mRNA-1273 without other blocking conditions; ctrl, healthy controls; px, patient.

The patient’s bone marrow T cells at diagnosis (**Figure 3A**), but not those of a control bone marrow (**Supplementary Figure S1**), and the patient’s peripheral blood immune cells during follow-up (**Figure 3B**), showed high spontaneous STAT3 phosphorylation (pSTAT3). *In vitro* stimulation of peripheral blood mononuclear cells of the patient with the mRNA-1273 vaccine resulted in distinct STAT3 protein phosphorylation in monocytes and T cells of the patient (**Figure 3C**). This was associated with mRNA-vaccine-induced IL-6 production *in vitro* (**Figure 3D**). pSTAT3 activation could be blocked by the anti-IL-6 blocking antibody siltuximab and the anti-IL-6 receptor blocker tocilizumab (TCZ) (**Figure 3E** and **Supplementary Figure S2**), indicating IL-6 mediated pSTAT3 upregulation. The mRNA vaccine also activated T cells IL-6-dependently (**Figure 3F**).

The IL-6/STAT3 pathway can be activated by toll-like receptors, including TLR-3 (15). Virus-derived double-stranded RNA (dsRNA), but also endogenous mRNA, can act as activators of TLR3 (23). The novel mRNA vaccines were modified to dampen RNA-mediated TLR3 activation by reducing dsRNA formation and introducing nucleotides with less inflammatory properties (16). However, even modified mRNA vaccines activate innate immunity *via* TLR3 (16). We confirmed TLR3 activation by stimulating peripheral blood mononuclear cells with mRNA-1273 resulting in increased phosphorylation of TLR3 (**Figure 3G**). Combined, our data suggest that the mRNA-1273 activates (innate) immune cells *via* TLR to secrete IL-6, subsequently resulting in STAT3 phosphorylation.

### Distinct immune activation following mRNA-1273 vs. AD26.COV2 stimulation

**Figure 4:**
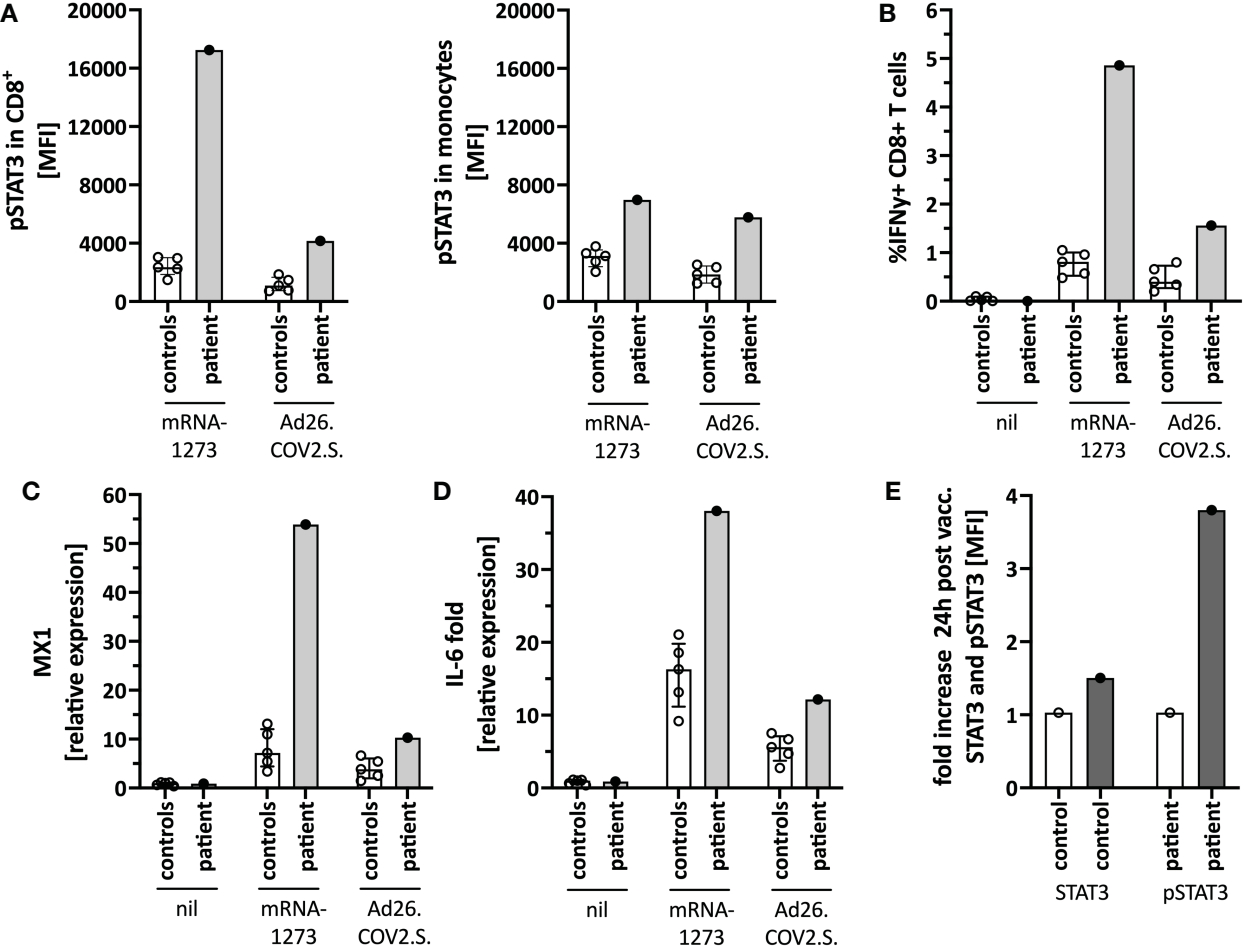
Immune activation by mRNA-1273 and Ad26.COV2.S vaccines. **(A)** STAT3 phosphorylation in CD8+T cells (left) and monocytes (right) following *in vitro* stimulation of peripheral blood immune cells with the mRNA-1273 vaccine or Ad26.COV2.S vector vaccine (pre-vaccine dose 2; i.e. different timepoint than in **Figure 1D**). **(B)** Vector vaccine stimulation induced lower T cell activation *in vitro* than the mRNA vaccine. **(C)** MX1 (=Interferon stimulated gene (ISG)) and **(D)** IL-6 induction upon *in vitro* stimulation with mRNA-1273 or Ad26.COV2.S is shown by qPCR. **(E)** Mild *in vivo* pSTAT3 activation 24 hours following vaccination with Ad26.COV2.S. Data is expressed as fold change of STAT3 or pSTAT3 activation 24h-post-vaccination normalized to an unvaccinated control. Nil= unstimulated condition= media only. ctrl= healthy controls; px= patient.

We next tested for differences in the *in vitro* immune activation between different COVID-19 vaccines, specifically mRNA-1273 and the adenoviral vector vaccine Ad26.COV2.S. We observed lower pSTAT3 activation (**Figure 4A**), IFNγ production in CD8^+^ T-cells (**Figure 4B**), as well as interferon-stimulated genes (ISG) (**Figure 4C**) and IL-6 (**Figure 4D**) induction when stimulating the patients’ PBMC with Ad26.COV2.S compared to mRNA-1273. Additionally, direct *ex vivo* stimulation of whole blood with the mRNA vaccine resulted in strong neutrophil degranulation in the patient, which could not be recapitulated after polyclonal T-cell stimulation with agonistic anti-CD3/28 antibodies, suggesting a T cell-independent effect (**Supplementary Figure S3**). Whether this *in vitro* observed degranulation occurs *in vivo* will need to be tested.

Based on the *in vitro* data, we speculated that a strong mRNA vaccine-mediated IL-6/STAT3 activation contributed to the adverse reaction by TLR-mediated innate immune cell activation and indirectly by T-cell activation. Since the vector vaccine showed weaker stimulation of these pathways *in vitro*, we completed her COVID-19 immunization series with a single dose of Ad26.COV2.S, which was well tolerated and caused only mild T-cell-intrinsic STAT3 phosphorylation (**Figure 4E**). Her blood counts, including neutrophil count, remained stable.

## Discussion

We provide evidence that the mRNA-1273 vaccine, and to a much lesser extent the Ad26.COV2.S vector vaccine induces STAT3 activation through TLR stimulation with the potential risk of exacerbating STAT3-dependent disease(s). We hypothesize that mRNA-vaccine-mediated STAT3 activation stimulated the T-LGL clone *via* IL-6 and activated innate cells in the reported case. Notably, clonal *STAT3* mutations have been directly linked to neutropenia in T-LGL (8). The increased IFNγ production in T cells following *in vitro* mRNA vaccine stimulation suggests that T cells may be activated either directly or *via* IL-6. Whether high IFNγ secretion contributed to the overactivation of innate immunity or neutropenia *in vivo* cannot be concluded based on the *in vitro* data and a single case. Interestingly, expansion of T cells causing progressive lymphadenopathy following mRNA vaccination has been reported in a patient with angioimmunoblastic T Cell lymphoma (24).

Based on the follow-up, the T-LGL clone persisted over months, but in the absence of symptoms and without the need for therapy, the T-LGL expansion may have been reactive and not reflect T-LGL leukemia.

The incidence of neutropenia following immunization with the mRNA vaccine BNT162b2 was reported as 2.6 per 100’000 vaccinated subjects. Notably, none of these cases was severe (defined as <500/ul neutrophils) and the incidence of neutropenia is about 40-times higher in patients with COVID-19 (88.4 per 100’000 cases) (25). In a cohort of 342 patients with inborn errors of immunity (IEI), one patient with common variable immunodeficiency developed severe neutropenia three days following BNT162b2 immunization. The patient required treatment with granulocyte stimulating factor and systemic corticosteroid therapy. Interestingly, the patient was subsequently diagnosed with T-LGL (26). To our knowledge, there are no other reports of severe neutropenia in T-LGL. Recently, good COVID-vaccine immunogenicity was demonstrated in a small cohort of mostly treated T-LGL patients (27). Safety data were not reported in this study.

Based on the *in vitro* findings, we completed immunization with the Ad26.COV2.S, which was well-tolerated. Thus, there was no rationale to re-expose her to an mRNA vaccine to test for causality. Our observation suggests that an alternative vaccination platform may be a personalized option for subjects experiencing STAT3-mediated adverse events following immunization with an mRNA vaccine.

## Data availability statement

The original contributions presented in the study are included in the article/**Supplementary Material**. The NGS sequencing data presented in the study are deposited in the European Nucleotide Archive (ENA) repository, accession numbers PRJEB58739 and ERP143807. Further inquiries can be directed to the corresponding author.

## Ethics statement

The studies involving human participants were reviewed and approved by ethics committee of Northwestern and Central Switzerland. The patients/participants provided their written informed consent to participate in this study. Written informed consent was obtained from the individual(s) for the publication of any potentially identifiable images or data included in this article.

## Author contributions

JRH designed and performed experiments, analyzed data, and drafted the manuscript. AT, IA, MR, TD, and SP collected and analyzed data and edited the manuscript. CTB designed and funded the study, analyzed the data, and drafted the manuscript. All authors contributed to the article and approved the submitted version.
