# Supplementary material for: Case Report: mRNA vaccination-mediated STAT3 overactivation with agranulocytosis and clonal T-LGL expansion
Source: Front Immunol. 2023 Feb 2;14:1087502. doi: 10.3389/fimmu.2023.1087502 (PMC9933345; doi:10.3389/fimmu.2023.1087502)
Supplement: Supplementary file 1 [file DataSheet_1.docx]

**Supplementary Data**

**Supplementary Methods:**

**Next Generation Sequencing**

The NGS library was prepared from DNA extracted from FFPE sections, using 20 ng DNA as input. For this purpose, the general Ampliseq™ Library Kit Plus (Thermo Fisher Scientific) protocol was adapted for use with the custom lymphoma panel v3, which includes 172 genes (131 with full coding coverage, 42 with hotspot-only coverage). This panel is an extension of the assay published in Vela V *et. al.* Mod Pathol 2021^1^ and includes STAT3 and STAT5b. The NGS library was quantified and diluted to 50 pM. It was then loaded onto an Ion 550™ chip using the Ion Chef™ instrument, and sequenced on the Ion GeneStudio™ S5 Prime System (Thermo Fisher Scientific). Raw data were processed automatically on the Torrent Server™ and aligned to the hg19 reference genome. Sequencing data were then uploaded in BAM format to the Ion Reporter™ Analysis Server for variant caller analysis using a custom workflow (Ion Reporter™ version 5.18).

**Whole blood assay – granulocytes**

Red blood cells were removed from whole blood using RBC lysis Buffer (from Biolegend). Lysed whole blood cells were stimulated with the covid vaccine [1:200] or CD3 and CD28 [2ug/ml] for 5, 15, 30, or 60 minutes at 37°C. Cells were stained for CD14 (FITC, 63D3), CD15 (PE, W6D3), CD16 (PeCy7, 3G8), and CD66b (APC, G10F5) during the last five minutes of stimulation at 37°C. Cells were immediately acquired on a BD LSRFortessa and analyzed using FlowJo v10.8.1.

**Supplementary Figure**

**
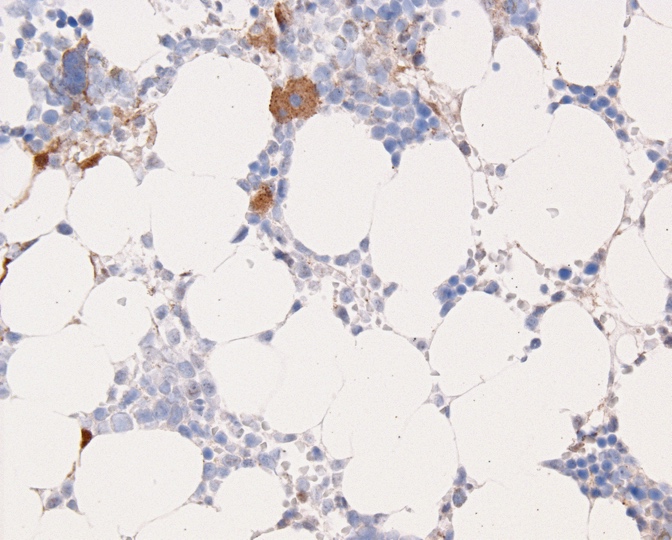
**

**Figure S1: Control staining for pSTAT3.** Reactive bone marrow (staging biopsy for a marginal zone B-cell lymphoma of the parotid) showing isolated pSTAT3 nuclear positive endothelial cells. Original magnification 400x.


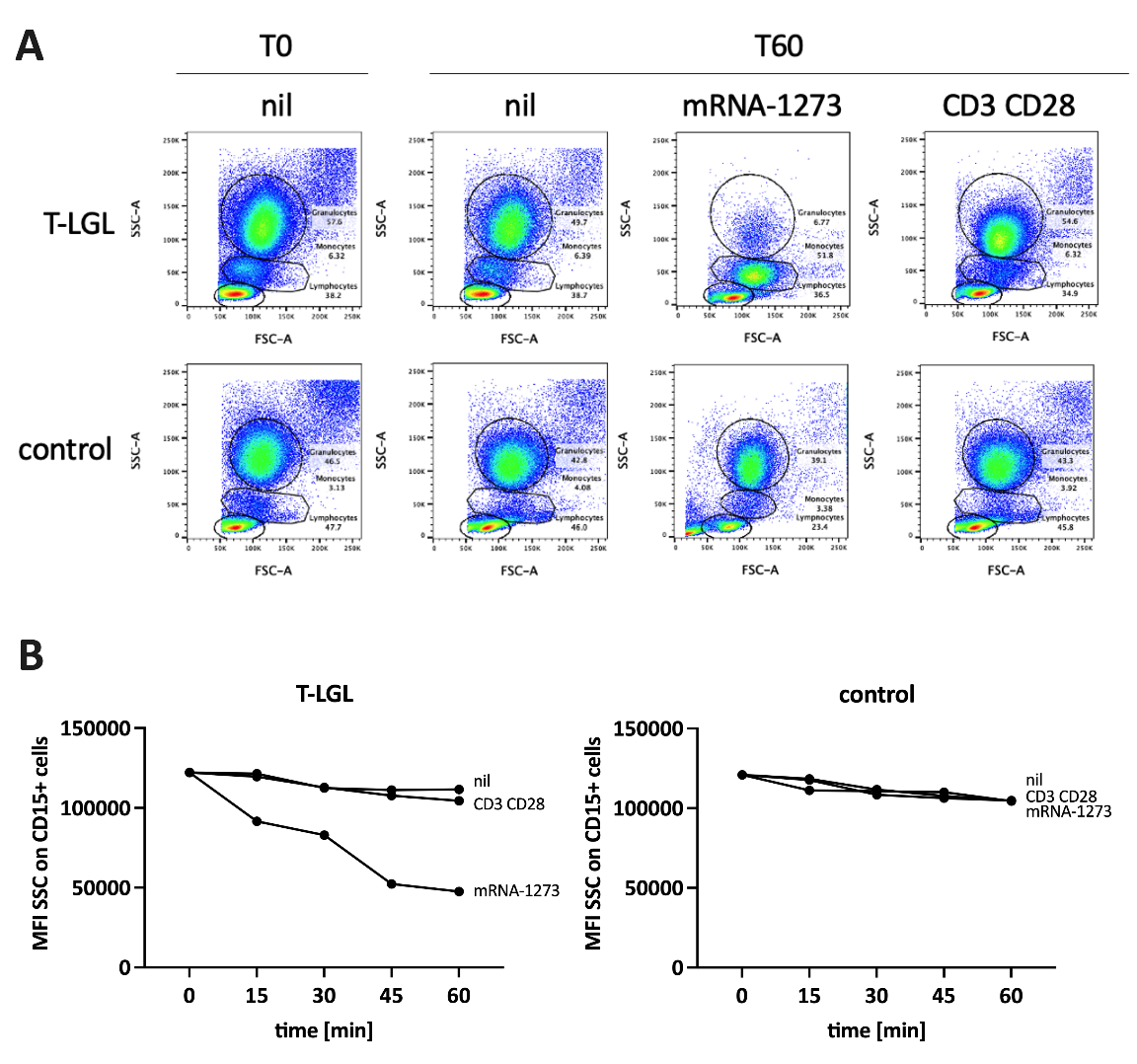


**Figure S1: *In vitro* effects of mRNA-1273 on neutrophils.**  (A) Flow cytometry dot plots are shown. The gates indicate the granulocyte, monocyte and lymphocyte gate, and the numbers indicate the %of total white blood counts in the respective gates. Direct *ex vivo* stimulation of whole blood of the patient with mRNA-1273 resulted in strong degranulation of neutrophils (indicated as a shift of the granulocyte population to lower side scatter median fluorescence intensity (MFI)) in the patient (bottom row) but not in a control (top row). Neutrophil degranulation could not be recapitulated by polyclonal T-cell stimulation with agonistic anti-CD3/28 antibodies, suggesting a T cell independent effect. T0= ex vivo; T60= 60 minutes after adding mRNA-1273. Nil=unstimulated; mRNA= mRNA-1273. (B) Summary graph indicate the time course of granulocyte degranulation (i.e., SSC MFI decline on CD15+ neutrophils) in the patient (left) and a control (right). Time indicates minutes after adding mRNA-1273 to whole blood.

**Figure S3: (A)** mRNA vaccine induced in vitro pSTAT3 is blocked by tocilizumab (TCZ, anti-IL6 receptor antibody). (B) mRNA stimulation induces T cell activation (IFNγ induction) via IL-6, indicated by the partial blocking by TCZ. IVIG was used as a non-IL-6 blocking control antibody.

**References**

1. Vela V, Juskevicius D, Prince SS, et al. Deciphering the genetic landscape of pulmonary lymphomas. *Mod Pathol*. Feb 2021;34(2):371-379. doi:10.1038/s41379-020-00660-2
